# The Rarity of Penetrating Ulcer with Intramural Hematoma of the Ascending Aorta

**DOI:** 10.21470/1678-9741-2020-0498

**Published:** 2022

**Authors:** Joshua Patino, Patrick T. Roughneen

**Affiliations:** 1 School of Medicine, Division of Cardiothoracic Surgery, University of Texas Medical Branch, Galveston, Texas, United States of America.; 2 Department of Surgery, Division of Cardiothoracic Surgery, University of Texas Medical Branch, Galveston, Texas, Unites States of America.

**Keywords:** Aorta, Hematoma, Atherosclerosis, Ulcer, Patient Discharge

## Abstract

We report a case of a 58-year-old man with multiple symptomatic penetrating atherosclerotic ulcers and concomitant intramural hematoma of the ascending aorta. The patient was successfully treated using a 24-mm Gelweave graft in the ascending aorta. He was discharged four days post operation and remains asymptomatic 14 months postoperatively. This case uniquely illustrates the rare entity of penetrating atherosclerotic ulcer with intramural hematoma, which presents in only 0.28% of all acute aortic syndrome cases.

**Table:** 

Abbreviations, acronyms & symbols	
AAS	= Acute aortic syndrome
CT	= Computed tomography
IMH	= Intramural hematoma
MRI	= Magnetic resonance imaging
PAU	= Penetrating atherosclerotic ulcer

## INTRODUCTION

Penetrating atherosclerotic ulcer (PAU) ulcerates through the internal elastic lamina of the aorta, resulting in intramural hematoma (IMH)^[1]^. PAU and IMH are both types of acute aortic syndrome (AAS), accounting for 2-7% and 10-25% of all AAS, respectively^[2]^. Several studies have characterized the aggressive nature of PAU and IMH, with high rupture rates (25-40%) for symptomatic cases^[3^,^4]^. Type A PAU with associated IMH represents 0.28% of all AAS cases^[5]^. We present this rarity in a 58-year-old man with AAS managed emergently with surgery.

## CASE PRESENTATION

**Fig. 1:**
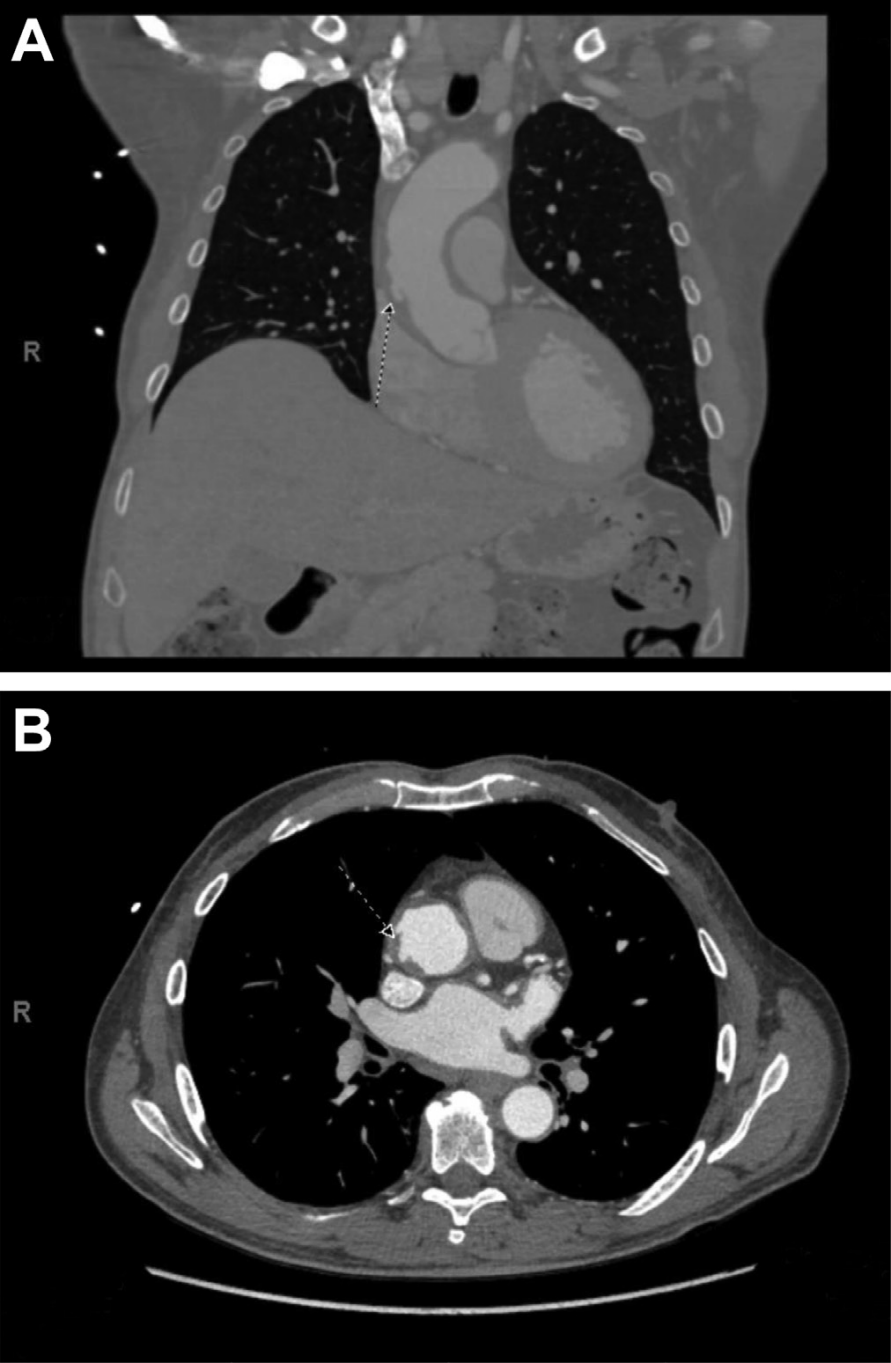
Computed tomographic angiograms in the A) coronal and B) axial views show intimal ulceration of the ascending aorta caused by a penetrating ulcer. Arrows indicate the outpouching.

The patient was hypertensive at presentation, with acute, left-sided, chest pain unresolved with intravenous esmolol and nicardipine. Computed tomography (CT) angiograms revealed thickening and irregularity of the ascending aorta with multiple intimal ulcerations and an IMH (Figure 1) requiring urgent surgery.

**Fig. 2:**
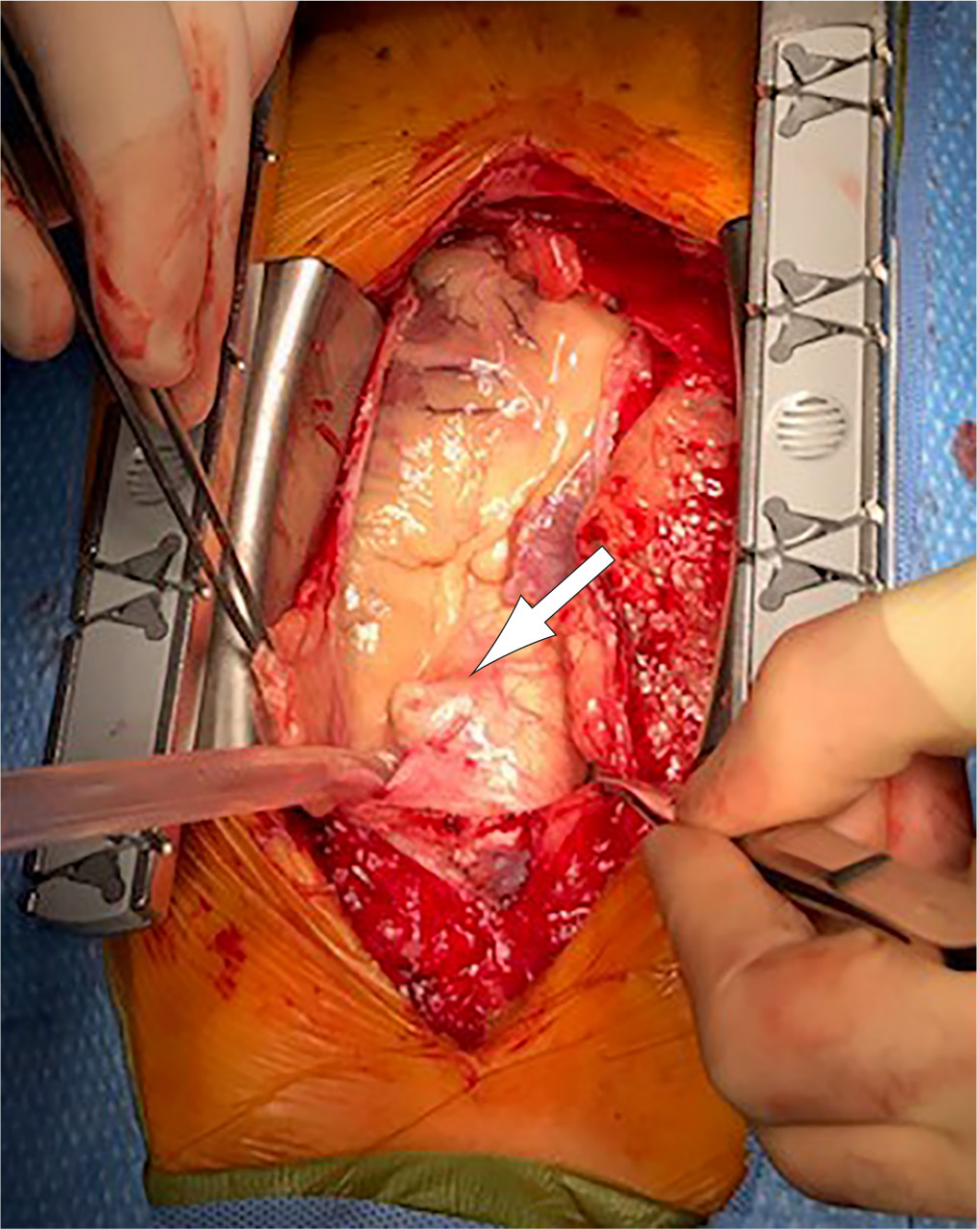
Gross intraoperative image of the ascending aorta demonstrates one of several outpouchings due to aortic ulceration with intramural hematoma. Arrow indicates the outpouching.

Following median sternotomy, significant outpouching of ascending aorta was visualized (Figure 2). Heparin administration and cardiopulmonary bypass were initiated, and systemic temperature was cooled to 32°C. Cardioplegic arrest was achieved utilizing 1800 mL of cold blood cardioplegia and an intramyocardial temperature of 15°C. During aortic cross-clamping, retrograde blood cardioplegia was administered every 10-15 minutes.

On aortotomy, multiple PAUs and an IMH were seen extending into the aortic wall. Following this, the diseased portion of the ascending aorta was resected. The aorta was then sized to a 24-mm Gelweave graft anastomosed using 4/0 Prolene (Ethicon; Somerville, New Jersey, United States of America) suture. The patient was rewarmed, weaned from bypass, and closed in a standard manner.

Twenty-four hours postoperatively, the patient developed cardiac tamponade. Mediastinal exploration was performed, and a large thrombus was evacuated. All surgical sites were hemostatic on inspection, and the patient was transferred back to the intensive care unit. He had an uneventful postoperative course and was discharged four days later.

## DISCUSSION

Extensive investigation of PAU was done by Shennan in 1934 and later established within the spectrum of AAS by Stanson et al.^[1^,^6]^. AAS constitutes variations of potentially life-threatening aortic conditions, including PAU, IMH, and classic dissection. These conditions present similarly with acute, intense chest pain described as tearing, ripping, or pulsating. However, these variants of AAS differ in their pathophysiology, natural history, and prognosis.

This case illustrates two important aspects of this disease process. First, the rarity of combined IMH and penetrating aorta in AAS, and second, the photographic depiction of an advanced case of an exfoliating penetrating ulcer with imminent rupture. This case also highlights the emergent nature of this relatively rare subset of AAS that is often underappreciated and undertreated.

PAU comprises 2-7% of all cases of AAS^[2]^ and is more commonly localized in the descending aorta than the ascending aorta or arch^[2^,^3]^. Patients are typically older with extensive atherosclerotic disease and hypertension. Symptomatic patients present with anterior chest or mid-scapular back pain. Imaging modalities include magnetic resonance imaging (MRI), contrast-CT scan, or transesophageal echo, revealing a focal irregular outpouching of the aortic wall^[3]^. PAU develops as an aortic ulcer penetrating through the internal elastic lamina and blood enters the media, resulting in a subadventitial IMH without intimal flap. The hematoma may extend further between the medial and adventitia layers, resulting in dissection^[7]^. If the adventitia is perforated, transmural aortic rupture occurs. The rupture rate of symptomatic PAU has been estimated as high as 40%^[2]^. Due to the high risk of aortic rupture with symptomatic PAU, medical therapy is aimed to reduce aortic sheering forces by decreasing cardiac contractility and blood pressure. Intravenous β-blockers and vasodilators are used to achieve a systolic blood pressure of 100-120 mmHg and heart rate of < 60 bpm. It was reported that medically managed symptomatic PAU patients progressed to rupture (38%), required surgical intervention (65%), or died during their hospital admission (15%)^[4]^. Surgery is therefore indicated to prevent or treat aortic rupture if pain persists despite medical therapy^[2]^. The current consensus is for surgical therapy with symptomatic Type A PAU and medical therapy and clinical/CTA surveillance for Type B PAU^[2]^. Surgical therapy involves either endovascular or open repair, although randomized controlled trials have yet to be performed to determine the optimal management of symptomatic PAU^[3]^.

Aortic IMH, described by Krukenberg in 1920, comprises 5-25% of AAS cases^[2]^. Similar to PAU, IMH is commonly found in the descending rather than ascending aorta^[1^,^5]^. Patients with symptomatic IMH have similar symptoms as those with PAU but are typically much older. The diagnostic imaging for IMH include CT and MRI, which reveal circular or crescentic thickening > 5 mm of the aortic wall with no evidence of blood flow^[3]^. Secondary to PAU formation, the pathogenesis of IMH is characterized by hemorrhage through the media without the presence of an intimal flap. Similar to PAU, symptomatic IMH has a significant risk of progression to classic aortic dissection (28-47%) or aortic rupture (20-45%)^[3]^. As there is a high mortality (40%) with symptomatic Type A IMH, emergent surgery is indicated^[3]^. Conversely, IMH of the descending aorta can be initially managed medically^[2]^.

## CONCLUSION

The case presented demonstrates the rare variant of AAS due to PAU with associated IMH and advocates an aggressive surgical approach for patients with symptomatic Type A PAU or IMH.

**Table:** 

Authors' roles & responsibilities	
JP	Substantial contributions to the analysis of data for the work; drafting the work and revising it critically for important intellectual content; agreement to be accountable for all aspects of the work in ensuring that questions related to the accuracy or integrity of any part of the work are appropriately investigated and resolved; final approval of the version to be published
PTR	Substantial contributions to the conception or design of the work and the acquisition, analysis, and interpretation of data for the work; drafting the work and revising it critically for important intellectual content; agreement to be accountable for all aspects of the work in ensuring that questions related to the accuracy or integrity of any part of the work are appropriately investigated and resolved; final approval of the version to be published
